# Disposable Electrochemical Sensor for Food Colorants Detection by Reduced Graphene Oxide and Methionine Film Modified Screen Printed Carbon Electrode

**DOI:** 10.3390/molecules26082312

**Published:** 2021-04-16

**Authors:** Chutimon Akkapinyo, Kittitat Subannajui, Yingyot Poo-arporn, Rungtiva P. Poo-arporn

**Affiliations:** 1 Biological Engineering Program, Faculty of Engineering, King Mongkut’s University of Technology Thonburi, Bangkok 10140, Thailand; chutimon.akk@gmail.com; 2 School of Materials Science and Innovation, Faculty of Science, Mahidol University, Bangkok 10400, Thailand; kittitat.sub@mahidol.ac.th; 3 Synchrotron Light Research Institute, 111 University Avenue, Nakhon Ratchasima 30000, Thailand; yingyot@slri.or.th

**Keywords:** azo dyes, amaranth, carminic acid, methionine, reduced graphene oxide, sunset yellow, tartrazine, sensor

## Abstract

A facile synthesis of reduced graphene oxide (rGO) and methionine film modified screen printed carbon electrode (rGO-methionine/SPCE) was proposed as a disposable sensor for determination of food colorants including amaranth, tartrazine, sunset yellow, and carminic acid. The fabrication process can be achieved in only 2 steps including drop-casting of rGO and electropolymerization of poly(L-methionine) film on SPCE. Surface morphology of modified electrode was studied by scanning electron microscopy (SEM). This work showed a successfully developed novel disposable sensor for detection of all 4 dyes as food colorants. The electrochemical behavior of all 4 food colorants were investigated on modified electrodes. The rGO-methionine/SPCE significantly enhanced catalytic activity of all 4 dyes. The pH value and accumulation time were optimized to obtain optimal condition of each colorant. Differential pulse voltammetry (DPV) was used for determination, and two linear detection ranges were observed for each dye. Linear detection ranges were found from 1 to 10 and 10 to 100 µM for amaranth, 1 to 10 and 10 to 85 µM for tartrazine, 1 to 10 and 10 to 50 µM for sunset yellow, and 1 to 20 and 20 to 60 µM for carminic acid. The limit of detection (LOD) was calculated at 57, 41, 48, and 36 nM for amaranth, tartrazine, sunset yellow, and carminic acid, respectively. In addition, the modified sensor also demonstrated high tolerance to interference substances, good repeatability, and high performance for real sample analysis.

## 1. Introduction

Recently, food colorants have demonstrated an outstanding role in improving food appearance and making them look more attractive. There are many of synthetic and natu-ral dyes that are used in the food industry. Nevertheless, excessive consumption of some colorants can lead to health risks. Therefore, the food law is required to control the amount of hazardous dyes in food.

**Scheme 1:**
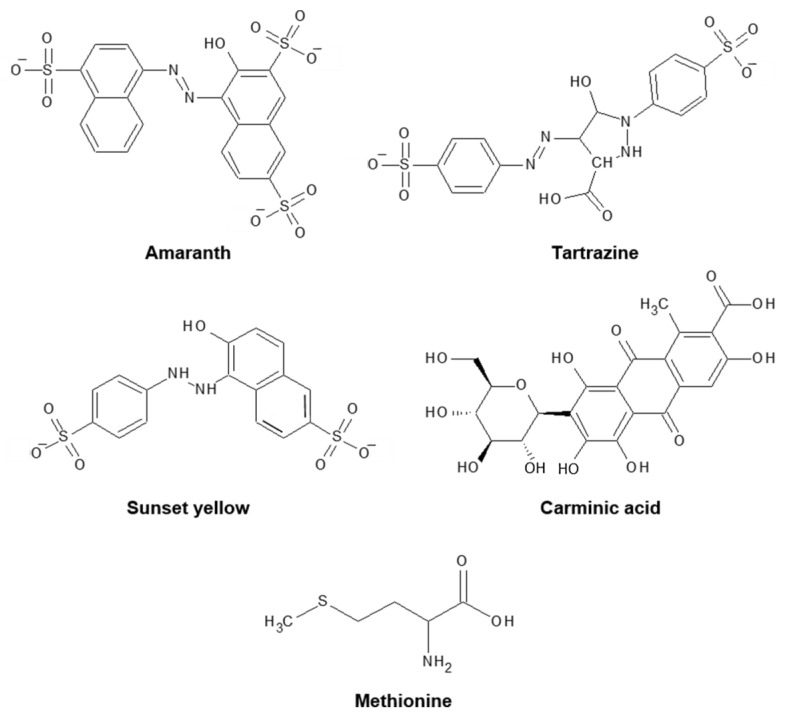
Chemical structure of amaranth, tartrazine, sunset yellow, carminic acid, and methionine.

Azo dyes are the most popular synthetic dye in the food industry. About 70% of syn-thetic food colorant belongs to azo dyes. In general, this dye is utilized for coloring of con-fectioneries, soft drinks, and alcoholic beverages. However, health effects were revealed for excessive consumption. The azo dye molecule can be cleaved into aromatic amines, which are suspected to be carcinogens and mutagens [1]. Moreover, superexcitation and hyper-activity in children were revealed for overconsumption [2]. To manage health risks, Euro-pean Food Safety Authority (EFSA) defined the acceptable daily intake (ADI) for each azo dye. For example, ADI was set at 0.15, 4.0, and 7.5 mg/kg body weight for amaranth (AM), sunset yellow (SY), and tartrazine (TZ), respectively [3,4,5]. The chemical structure of ama-ranth, tartrazine, and sunset yellow are illustrated in Scheme 1. Given that the molecule contains azo group (–N = N–), it is possible to be reduced by electrochemical reduction. Moreover, there is a hydroxyl group on their chemical structure, thus they can be oxidized as well [6].

As the consumer requires foods to be as natural as possible, natural dyes are an al-ternative way of coloring food. Carminic acid (CA) is a red shade natural dye obtained from dried bodies of insects, Dactylopius coccus. The chemical structure of carminic acid is illustrated in Scheme 1. Its chemical structure consists of a glucose and an anthraqui-none [7]. In comparison with azo dyes, carminic acid does not exhibit carcinogenic and toxic properties [8]. Given that carminic acid is produced from insects, there are insect proteins left over from the manufacturing process. These proteins can trigger acute hyper-sensitivity reactions and cause severe anaphylactic reactions [8]. Moreover, due to the low molecular size of carminic acid, it can act as a hapten and trigger immune response when it combines with large carrier molecules such as proteins [9]. Consequently, EFSA defined ADI of carminic acid at 2.5 mg/kg body weight and introduced the appropriate purifica-tion to reduce allergens as much as possible [8]. Therefore, an effective and reliable method is required for detecting those food colorants.

Due to high sensitivity, high specificity, low cost, short time analysis, and simplicity, electrochemical methods were widely proposed for food dyes determination instead of conventional methods, i.e., spectrophotometry [10,11], chromatography [12], and capillary electrophoresis [13]. According to azo and hydroxyl group of azo dyes, direct voltammet-ric determination is divided into 2 categories including cathodic reduction and anodic oxidation. However, azo dyes determination by azo reduction attracts less attention than hydroxyl oxidation due to the problem of removal of dissolved oxygen [6]. The reduction of dissolved oxygen can alter the cathodic current response of azo dyes. To improve sensitiv-ity and specificity, nanomaterials or chemicals were modified on several types of electrode.

Graphene is a single layer of carbon atoms arranged in a hexagonal lattice. This structure confers fascinating properties to graphene including large surface area and high electrical conductivity [14]. Therefore, graphene and its derivatives as a hybrid material have been widely used in electrochemical sensor field.

Given that amino acids contain amine (–NH_2_) and carboxyl (–COOH) functional groups, they are able to form conductive polymer by electropolymerization. Beside electrocatalytic activity improvement, poly(amino acid) film also provides more active sites for target analytes resulting in sensitivity and selectivity enhancement of the sensor [15]. Therefore, poly(amino acid) has received great attention to modify on electrode surface for sensitivity improvement. Various substances have been determined by amino acid based electrochemical sensor, for example, butylated hydrox-yanisole (BHA) [16], simultaneous detection of uric acid, xanthine and hypoxanthine [17], and epinephrine [18]. Moreover, there is a cooperation between poly(amino acid) film and other nanomaterials to increase electrode’s sensitivity, for example, methionine/gold na-noparticles modified glassy carbon electrode (GCE) for hydroquinone determination. With high surface area and strong adsorptive ability of methionine/gold nanoparticles film, the accumulation of hydroquinone on the electrode was improved [19]. Poly(l-arginine)/graphene composite film modified GCE was proposed for simultaneous determination of uric acid, xanthine, and hypoxanthine. With the catalytic property of poly(l-arginine) and excellent electric conductivity of graphene, the electrochemical signal was amplified [20]. In addition, poly(amino acid) film was used for azo dyes determination as well [21,22,23,24,25,26]. On the other hand, only a few reports based on poly(amino acid) film was proposed for electrochemical determination of carmine [21]. However, previous poly(amino acid) film based electrochemical sensor for azo dyes determination is still em-ployed on traditional electrodes, i.e., GCE, carbon paste electrode or even pencil graphite electrode. Although these types of electrodes exhibit high sensitivity, they still lack the ca-pability for on-site detection. Due to the limitation of high cost, they need to be cleaned for reuse. This stint makes traditional electrodes inappropriate for mass production. Screen printed carbon electrode (SPCE) was proposed to break these limitations. SPCE demon-strates various advantages such as low fabrication cost, variable configuration, and being able to be produced for large scale. Thus, SPCE is appropriate to use for on-site detection. However, to the best of our knowledge, there is no report of electrochemical sensors based on reduced graphene oxide (rGO) and methionine film modified SPCE for azo dyes and carminic acid determination. Therefore, this is the first developed novel disposable sensor for detection of 4 dyes; amaranth, tartrazine, sunset yellow, and carminic acid.

This work aims to develop a disposable sensor for food colorants detection. The chemical structure of methionine presents in Scheme 1. The synergistic effect of rGO and methio-nine film are proposed for sensitivity improvement of SPCE. Poly(l-methionine) film and rGO were modified on SPCE for determination of carminic acid and 3 azo dyes (amaranth, tartrazine, and sunset yellow). With synergistic effect, the modified electrode demonstrated good catalytic activity to target analytes. Moreover, the modified SPCE demonstrated good performance for real sample analysis.

## 2. Results and Discussion

### 2.1. Morphology of Modified Electrode

**Figure 1:**
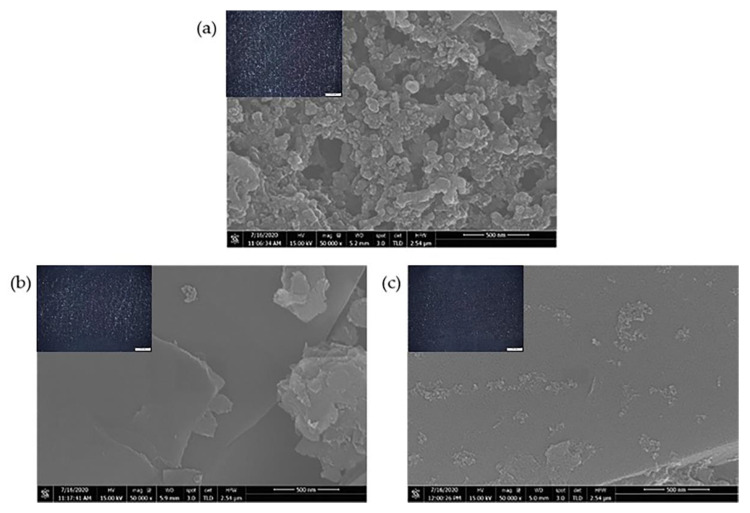
SEM images of bare SPCE (**a**), rGO/SPCE (**b**), and rGO-methionine/SPCE (**c**). The insets of (**a**–**c**) are the stereomicroscopic at 10× magnificent of bare SPCE rGO/SPCE and rGO-methionine/SPCE, respectively.

The morphology of bare SPCE, rGO/SPCE, and rGO-Methionine/SPCE were charac-terized by SEM as demonstrated in Figure 1a–c. The bare SPCE presented rough surface while smooth surface was observed on rGO/SPCE. The smooth surface is the layer of rGO that covered the SPCE. Thereafter, polymeric layer was observed on the layer of rGO after electrochemical polymerization of methionine film on rGO/SPCE. SEM result demonstrat-ed successful deposition of rGO and electropolymerization of methionine on SPCE. The insets of Figure 1a–c represent the image of bare SPCE, rGO/SPCE, and rGO-methionine/SPCE taking by light microscope. Bare SPCE demonstrated rough surface which was different to rGO/SPCE and rGO-methionine/SPCE. With the modification of rGO, the smoother surface was observed on rGO/SPCE and rGO-methionine/SPCE. More-over, with the methionine film covering, the fine surface demonstrated on rGO-methionine/SPCE. Thus, the results from microscopic image can ensure the success-ful modification of rGO and methionine film on SPCE.

### 2.2. Electrochemical Characterization of Electrode Modification

**Figure 2:**
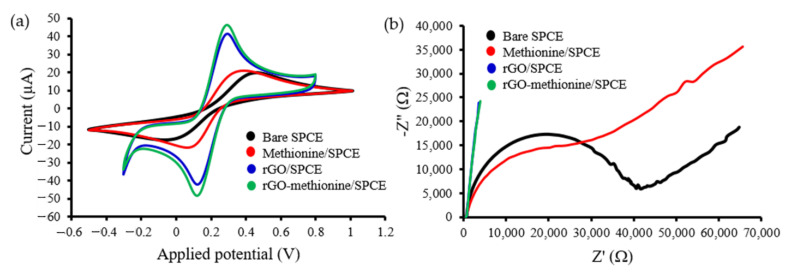
Electrochemical characterization of electrode modification by CV at scan rate 50 mV/s (**a**) and EIS (**b**).

To study the effect of rGO and methionine on SPCE and characterize the modification process, cyclic voltammetry (CV) and electrochemical impedance spectroscopy (EIS) were used to investigate each step of SPCE modification. A 5 mM of ferricyanide/ferrocianyde redox couple (Fe(CN)_6_^3^^−/4^^−^) containing 0.1 M KCl was used as electrolyte. The cyclic voltammograms of rGO/SPCE, methionine/SPCE, and rGO-methionine/SPCE were compared with bare SPCE at scan rate of 50 mV/s as illustrated in Figure 2a. The current response was the signal of the redox probe. The obtained current response increased from bare SPCE, methionine/SPCE, rGO/SPCE, and rGO-methionine/SPCE, respectively. Given that the effect of high surface area of rGO can accelerate electron transfer [14], the current response was significantly increased on rGO/SPCE. The obtained current response of rGO/SPCE increased almost 5 times to bare SPCE. However, since methionine film is a barely conductive material, the catalytic current increased slightly after modification on bare SPCE and rGO/SPCE. Moreover, the result demonstrated the same tendency with EIS as depicted in Figure 2b. EIS was performed at amplitude of 10 mV. The charge transfer re-sistance (Rct) of bare SPCE and methionine/SPCE were interpreted by Randle’s equivalent circuit. Bare SPCE exhibited the largest Rct (38.49 ± 1.74 kΩ) as a result of the poor conduc-tivity of SPCE. For methionine/SPCE, the semicircle can still be observed, but it was smaller than bare SPCE. The Rct was found to be 29.79 ± 2.22 kΩ. However, the semicircle was not revealed on both rGO and rGO-methionine/SPCE. This can be attributed to rGO, which can improve the conductivity of SPCE. Moreover, we can prove that rGO and methionine film was successfully modified on SPCE by the changed current response and Rct of each step of modification.

### 2.3. Electrochemical Behavior of Amaranth, Tartrazine, Sunset Yellow and Carminic Acid on Modified Electrode

**Figure 3:**
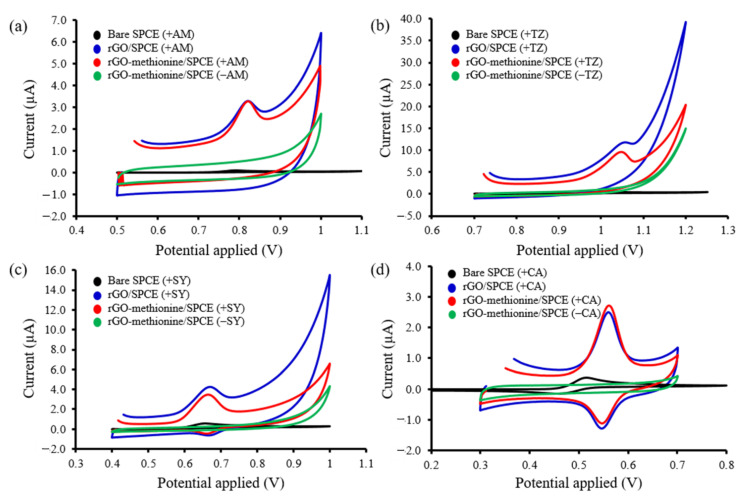
CV curves of 0 and 10 μM amaranth (**a**), 0 and 50 µM Tartrazine (**b**), 0 and 50 µM sunset yellow (**c**), 0 and 50 µM of carminic acid (**d**) on bare SPCE (black), rGO/SPCE (blue), and rGO-methionine modified SPCE (red) at scan rate 20 mv/s.

The electrochemical behavior of each dye was tested separately on rGO-methionine/SPCE in comparison with bare SPCE and rGO/SPCE to investigate performance of modified sensor. CV was used to study the electrochemical behavior of all 4 dyes. Cyclic voltammograms of 0 and 10 µM of amaranth, 0 and 50 µM of tartrazine, 0 and 50 µM of sunset yellow, and 0 and 50 µM of carminic acid were recorded as demonstrated in Figure 3a–d, respectively. The results demonstrated that catalytic current response was observed only when the detected dyes presented in the samples. Thus, the current response was the signal of the detected dyes. The oxidative current responses of each dye on bare SPCE, rGO/SPCE, and rGO-methionine/SPCE are presented in Table 1. For amaranth, acetate buffer pH 4.0 was used as the supporting electrolyte. The anodic peak of amaranth was observed at about 0.8 V, and no cathodic peak was observed on all of 3 different modified electrodes, as shown in Figure 3a. Only anodic peak was also observed for tartrazine. The oxidation peak appeared at a potential of about 1.05 V in citrate-phosphate buffer pH 3.0 (Figure 3b). For sunset yellow, voltammograms were recorded by using phosphate buffer pH 7.0, while carminic acid used citrate phosphate buffer pH 3.0 as electrolyte. Both of sunset yellow and carminic acid demonstrated a pair of redox peak as illustrated in Figure 3c,d, respectively.

All 4 dyes represented the consistent result. Bare SPCE demonstrated small electrochemical current response indicating weak catalytic activity of dyes on bare electrode, while the rGO/SPCE significantly enhanced the electrochemical response of all 4 dyes. With the high conductive property, the rGO film can help to facilitate electron transfer rate on electrode. This phenomenon affects the improvement of sensitivity [14]. Moreover, the layer of poly(L-methionine) film has a good catalytic effect due to its active groups, such as amino group and carboxyl group [17,25]. In addition, the result showed that methionine film decreased the background current of rGO. The well-defined peak was observed on rGO-methionine/SPCE. With the synergistic effect between rGO and poly(L-methionine) film, the catalytic activity of amaranth, tartrazine, sunset yellow, and carminic acid were significantly enhanced on rGO-methionine/SPCE. 

### 2.4. Effect of pH of Electrolyte

**Figure 4:**
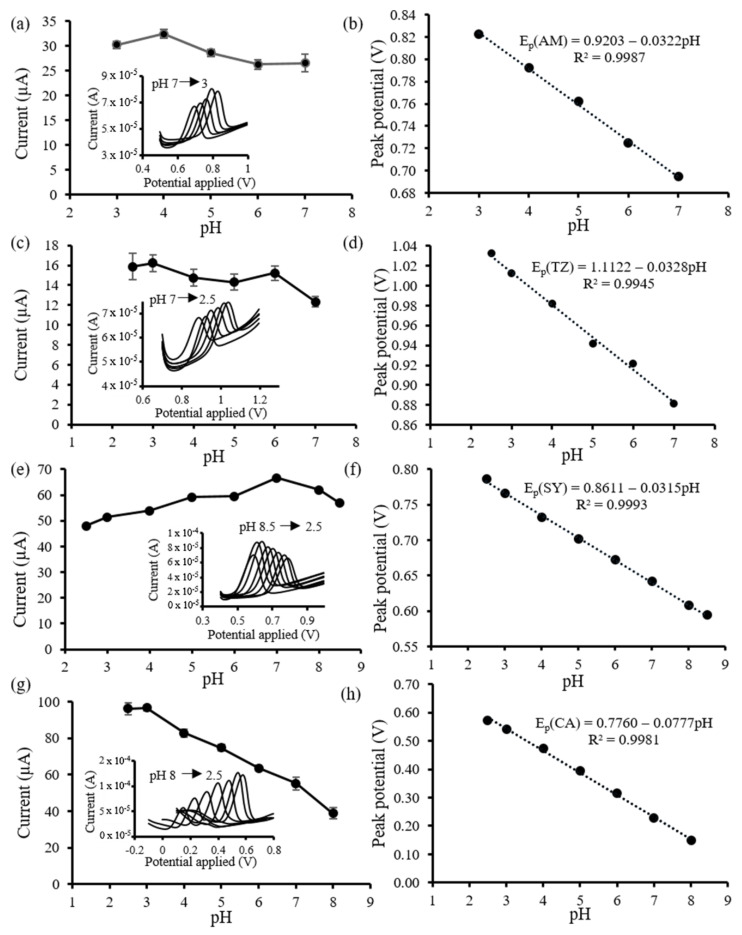
Representation of pH optimization for amaranth (**a**), tartrazine (**c**), sunset yellow (**e**), and carminic acid (**g**). The insets illustrate differential pulse voltammograms at different pH values. The (**b**,**d**,**f**,**h**) represent linear regression between pH and anodic peak potential of amaranth, tartrazine, sunset yellow, and carminic acid, respectively.

The appropriate electrolyte and its pH is the most important factor required to obtain the best catalytic current response, therefore various types of electrolytes at different pH values, including citrate-phosphate buffer at pH 2.5 and 3.0, acetate buffer at pH 4.0 and 5.0, and phosphate buffer at pH 6.0, 7.0, and 8.0, were investigated. The effect of pH on the catalytic activity of 50 μM amaranth, tartrazine, sunset yellow, and carminic acid was studied on rGO-methionine/SPCE by DPV. The results are illustrated in Figure 4a–h. Catalytic activities of amaranth, tartrazine, sunset yellow, and carminic acid were pH dependent. The anodic peak potential of all 4 dyes shifted to a negative direction with further increase in pH. This evidence can be attributed to the participation of protons in electrode reaction. In addition, the relationship between peak potential (Ep) and pH value was investigated. The linear regressions were demonstrated as E(AM) = 0.9203–0.0322 pH, R^2^ = 0.9987 for amaranth, E(TZ) = 1.1122–0.0328 pH, R^2^ = 0.9945 for tartrazine, E(SY) = 0.8611–0.0315 pH, R^2^ = 0.9993 for sunset yellow, and E(CA) = 0.7760–0.0777 pH, R^2^ = 0.9981 for carminic acid. The slope values at 0.0322, 0.0328, and 0.0315 of amaranth, tartrazine, and sunset yellow were close to half of theoretical value (0.059 V/pH). Therefore, the ratio between proton and electron in the reaction was equal to 1:2. This ratio is line with previous reports of amaranth [27,28,29], tartrazine [30,31], and sunset yellow [25,32]. The electrochemical mechanism of amaranth, sunset yellow and tartrazine may be summarized as follow [33]:

Dye − 2e^−^1H^+^→Dye (ox)

where dye refers to amaranth, sunset yellow, and tartrazine. On the other hand, the slope value between E(CA) vs. pH was almost close to the theoretical value, indicating that the number of protons was equal to the number of electrons involved in the reaction. The oxidation of carminic acid was based on hydroquinone [34], and can be summarized as follows:

Carminic acid − 22^−^ − 2H^+^ → Carminic acid (ox)

To achieve the best condition, the pH value of the highest oxidative current response was chosen for further experiments. Thus, acetate buffer pH 4.0 was chosen for determination of amaranth, while citrate phosphate buffer pH 3.0 was applied for tartrazine and carminic acid. For sunset yellow, the highest current response was obtained from phosphate buffer pH 7.0, therefore it was chosen for determination of sunset yellow.

### 2.5. Effect of Scan Rate

**Figure 5:**
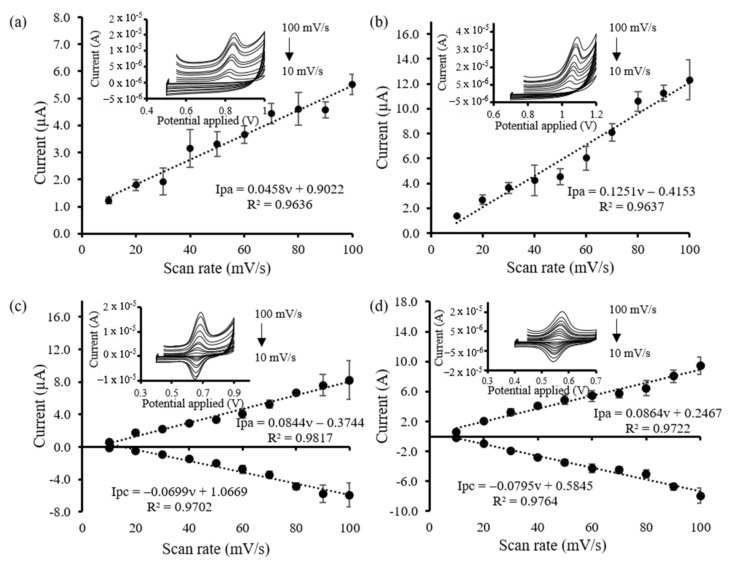
Linear relationship between scan rate and current response of amaranth (**a**), tartrazine (**b**), sunset yellow (**c**), and carminic acid (**d**). Insets represent obtained cyclic voltammograms at different scan rates.

The influence of scan rate on catalytic activity of amaranth, tartrazine, sunset yellow, and carminic acid were investigated on rGO-methionine/SPCE by CV at scan rate of 10 to 100 mV/s. The obtained optimal pH value from previous section (Section 2.4) was applied for each dyes. Similar result was observed from all 4 dyes as shown in Figure 5a–d. The catalytic current response increased corresponding to increasing scan rate from 10 to 100 mV/s. The linear equations were obtained as Ipa = 0.0458v + 0.9022, R2 = 0.9636 for amaranth, Ipa = 0.1251v − 0.4153, R^2^ = 0.9637 for tartrazine, Ipa = 0.0844v − 0.3744, R^2^ = 0.9817 and Ipc = −0.0699v + 1.0699, R^2^ = 0.9702 for sunset yellow, and Ipa = 0.0864v + 0.2467, R^2^ = 0.9722 and Ipc = −0.0795v + 0.5845, R^2^ = 0.9764 for carminic acid. The results demonstrated a linear relationship between catalytic current response and scan rate suggesting that electrochemical reactions of amaranth, tartrazine, sunset yellow, and carminic acid on rGO-methionine/SPCE were controlled by adsorption-controlled process.

### 2.6. Effect of Accumulation Time

**Figure 6:**
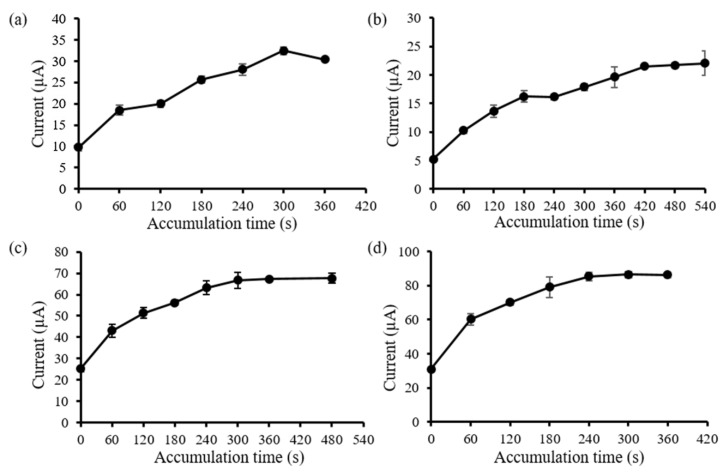
Oxidative current response of 50 µM of amaranth (**a**), tartrazine (**b**), sunset yellow (**c**), and carminic acid (**d**) at different accumulation time.

Given that the catalytic activities of amaranth, tartrazine, sunset yellow, and carminic acid are controlled by adsorption, the effect of accumulation time on rGO-methionine/SPCE was evaluated by DPV. Oxidative current response of 50 µM of each dye was evaluated in this experiment. All 4 dyes demonstrated similar results, where catalytic current response increased until it reached saturation time and was stable or dropped slightly after further increase in time, as demonstrated in Figure 6a–d. This may be the result of saturation of amaranth, tartrazine, sunset yellow, and carminic acid molecules on the modified electrode [35]. To accomplish the highest response and minimize the detection time, the lowest accumulation time at highest oxidative current was chosen for determination of each dye. Consequently, accumulation time at 300 s was applied for amaranth and sunset yellow, 420 s for tartrazine, and 240 s for carminic acid determination.

### 2.7. Determination of Linear Range and Detection Limit

**Figure 7:**
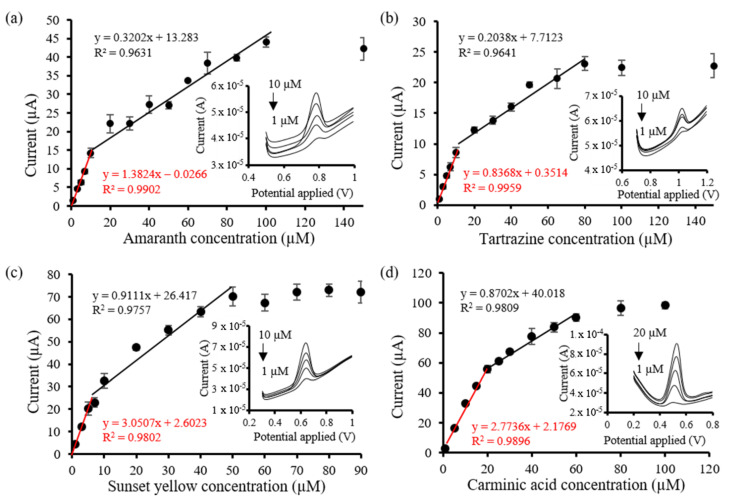
The linear relationship between the oxidative current response and concentration of amaranth (**a**), tartrazine (**b**), sunset yellow (**c**), and carminic acid (**d**). The insets represent the differential pulse voltammograms at the first linear detection range of each dye.

DPV was utilized to determine the linear detection range of amaranth, tartrazine, sunset yellow, and carminic acid. Step potential at 0.01 V and modulation amplitude at 0.1 V were applied for all determinations. Each concentration was tested in triplicate. The two linear detection ranges were observed. For amaranth, the detection was performed under potential from 0.5 to 1 V in acetate buffer pH 4.0 with accumulation time at 300 s. The current response increased proportionally with concentration of amaranth. Two linear detection ranges were obtained at 1 to 10 and 10 to 100 µM of amaranth, as shown in Figure 7a. The equations can be expressed as y = 1.3824x − 0.0266, R^2^ = 0.9902, and y = 0.3202x + 13.283, R^2^ = 0.9631. The limit of detection (LOD) was calculated from 3 times the standard deviation of the blank (n = 10) divided by the slope from the linear range, thus the LOD was obtained at 57 nM.

For tartrazine, potential range of 0.7 to 1.2 V, citrate phosphate buffer pH 3.0, and accumulation time of 420 s were used for tartrazine determination. Oxidative current also increased proportionally with tartrazine concentration. Figure 7b demonstrates linear detection ranges of tartrazine at 1 to 10 and 10 to 85 µM. The linear equations were obtained as y = 0.8368x + 0.3514, R^2^ = 0.9959, and y = 0.2038x + 7.7123, R^2^ = 0.9641. LOD was calculated to be 41 nM.

For sunset yellow, the determination was done under potential range of 0.3 to 1 V in phosphate buffer pH 7.0 with accumulation time at 300 s. Current response increased proportionally with increase in sunset yellow concentration and exhibited two detection ranges at 1 to 10 and 10 to 50 µM as shown in Figure 7c. The linear equations were expressed as y = 3.0507x + 2.6023, R^2^ = 0.9802, and y = 0.9111x + 26.417, R^2^ = 0.9757. LOD of 48 nM was obtained for sunset yellow determination.

To determine carminic acid concentration, a potential window from 0.2 to 0.8 V was applied. The electrochemical determination was done under the condition of citrate phosphate buffer pH 3.0 and accumulation time at 240 s. Two linear ranges were observed as well as the other 3 dyes, but the concentration range was different, as shown in Figure 7d. The first linear range was observed from 1 to 20 µM, and the second linear range was observed from 20 to 60 µM of carminic acid. Linear equations were demonstrated as y = 2.7736x + 2.1769, R^2^ = 0.9896, and y = 0.8702x + 40.018, R^2^ = 0.9809 while LOD was calculated as 36 nM.

However, the optimization conditions were performed at 50 µM of detecting dyes which locate in the second linear range of all 4 detecting dyes. The change of slope of peak current versus concentration plot might affect the change in reaction mechanism, number of electrons involved, and accumulation time. Thus, the proposed conditions were the optimal condition for only the linear detection range of 50 µM. Type of reaction mechanism and number of electron transfer were reported for linear detection range of 50 µM as well.

The performance comparison of previous reports and the modified sensor is shown in Table 2. The modified electrode showed excellent and recovery range of detection in all dyes. EFSA has regulated the maximum permitted level (MPL) of use of amaranth at 30 mg/L for fish roe, aperitif wines, and spirit drinks, including products with less than 15% alcohol by volume [36]. MPL at 50 and 100 mg/L of non-alcoholic flavored drinks was defined for sunset yellow [37] and tartrazine [5], respectively. For carminic acid, MPL was set ranging from 50 to 500 mg/kg in 58 food categories [8]. The obtained LOD of amaranth, tartrazine, sunset yellow, and carminic acid were below the MPL which was regulated by EFSA. Thus, the modified sensor was acceptable. In addition, as the modified sensor was fabricated by using SPCE as based electrode and decoration with inexpensive materials and simple method, it gained the advantages of simple preparation, low cost and short analysis time compared with previous reports. Moreover, given that SPCE is a disposable electrode, the modified electrode can be used as a portable device, which is convenient for on-site detection.

### 2.8. Interference Test

In general, soft drinks, beverages, or other confectionaries contain several ingredients, thus there is a need to be concerned about the effect of interference on the performance of the sensor. To study the interference of other substances, the amaranth, tartrazine, sunset yellow, and carminic acid at 5 and 50 µM were tested with common substances in beverages at various concentrations. Effect of interference was studied by DPV, and the results of interference substances testing with dye concentration at 5 and 50 µM are presented in Table 3 and Table 4, respectively. Current responses of with and without interference substance were compared and reported in terms of variance. The results demonstrated that 1000-fold concentration of glucose and sucrose, 100-fold concentration of NaCl and KCl, and 10-fold concentration of glycine, ascorbic acid, and citric acid did not significantly affect the determination of all 4 dyes on both concentrations (5 and 50 µM). The percentage error was obtained below ±5% which indicated good selectivity for amaranth, tartrazine, sunset yellow, and carminic acid.

### 2.9. Real Sample Analysis

To study the performance of the modified sensor, it was tested with real samples. The standard addition method was used to determine concentration of amaranth, tartrazine, sunset yellow, and carminic acid in real sample. The rGO-methionine/SPCE was evaluated with 2 groups of sample with and without target dye. Sprite no sugar (lemon-lime flavored soft drink) was tested as a sample without target dyes. Spy wine cooler (red) was tested as a sample containing target amaranth dye. 100 Plus lemon lime flavored carbonated drink and Sponsor active vitamin C lemon lime flavored were evaluated for tartrazine detection. Two orange flavor soft drinks, Mirinda and Fanta, were tested for sunset yellow determination. Betagen (strawberry flavored probiotic milk beverage) and Nestlé Eskimo Ice-Cream (Nomyen flavored ice cream with milk flavored white compound coating) were tested for carminic acid evaluation. The results presented in Table 5, Table 6, Table 7 and Table 8 are for amaranth, tartrazine, sunset yellow, and carminic acid, respectively. The great percentage recovery obtained for all 4 dyes indicated great performance, high accuracy, and reliability of the modified sensor for real sample analysis.

Moreover, UV-Vis spectrophotometer, was tested to confirm the performance of rGO-methionine/SPCE. Unfortunately, the real samples containing the interested dyes show high interference when measured using UV-Vis absorbance measurement. The other substances as well as its translucence interfered the absorbance. Therefore, only 1 sample (Sprite) with the spiked dye solution can be compared by this method. The standard addition method was also used for dyes determination. The result of UV-Vis measurement shows in the parentheses in Table 5, Table 6, Table 7 and Table 8. The result showed satisfying %recovery for all 4 detecting dyes. Moreover, the %recovery from UV-Vis measurement and the rGO-methionine/SPCE was about the same, indicating that the modified electrode was reliable for amaranth, tartrazine, sunset yellow, and carminic acid determination.

### 2.10. Reproducibility Test

To study reproducibility of modified sensor, the rGO-methionine/SPCE was tested with 50 µM of amaranth, tartrazine, sunset yellow, and carminic acid on 50 independent modified electrodes. The repeatability performance was reported in terms of the percent of relative standard deviation (%RSD). The %RSD was found to be 8.98, 6.49, 8.91, and 4.17 for amaranth, tartrazine, sunset yellow, and carmnic acid, respectively. The %RSD of ama-ranth, tartrazine, and sunset yellow was over 5%, but below 10%, indicating the slight ef-fect of independent preparation.

## 3. Materials and Methods

### 3.1. Reagents and Apparatus

Graphene oxide (GO), l-methionine, amaranth, sunset yellow, and tartrazine were purchased from Sigma-Aldrich Pte. Ltd. (Singapore). Carminic acid was obtained from Tokyo Chemical Industry (TCI) (Tokyo, Japan). All other chemicals were of analytical grade. All supporting electrolytes were prepared with milliQ water. Citrate-Phosphate buffer pH 2.5 and 3.0 were prepared from adjusting solution between 0.1 M citric acid and 0.2 M Na_2_HPO_4_. Acetate buffer pH 4.0 and 5.0 were prepared from mixing solution of 0.1 M acetic acid and 0.1 M of sodium acetate. Phosphate buffer solution (PBS) pH 6.0–8.0 were prepared from 0.1 M Na_2_HPO_4_ and 0.1 M NaH_2_PO_4_. All electrolytes were prepared using 0.1 M KCl and purged with nitrogen gas for at least 30 min to eliminate dissolved oxygen.

All the electrochemical measurements including CV, EIS, and DPV were performed on Autolab PGSTAT128N with NOVA software version 1.11. Screen printed carbon electrode (SPCE) was purchased from Quasence. Co, Ltd. (Bangkok, Thailand). The working elec-trode’s area was 3 mm^2^. A conventional three-electrode system containing the modified SPCE was used as working electrode, Ag/AgCl as reference electrode and platinum wire as counter electrode. UV-Vis spectrophotometer was used as a standard method for compar-ing in real sample analysis. The detecting samples for amaranth, tartrazine, sunset yel-low, and carminic acid were measured at the wavelength of 520, 426, 485, and 500 nm, respectively.

### 3.2. Synthesis of rGO

Reduced graphene oxide was prepared by glucose reduction [51,52]. Firstly, 10 mg of graphene oxide (GO) and 40 mg of glucose were dispersed in 10 mL of deionized (DI) water. The mixed solution was stirred for 30 min at 95 °C. Thereafter, 100 µL of ammonium hy-droxide (~25% NH_3_ basis) was added by dropping. Then, the mixed solution was stirred for 1 h at 95 °C. The rGO was collected by filtration on nitrocellulose membrane with 0.45 μm pore size and washed with DI water until filtrated solution attained a pH of 7.0. The synthesized rGO was separated from membrane by sonication in DI water. Thereafter, the dispersed rGO was collected by centrifugation. The precipitated rGO was dried overnight at 37 °C and kept in dry condition.

### 3.3. Preparation of rGO-Methionine Modified SPCE

**Scheme 2:**
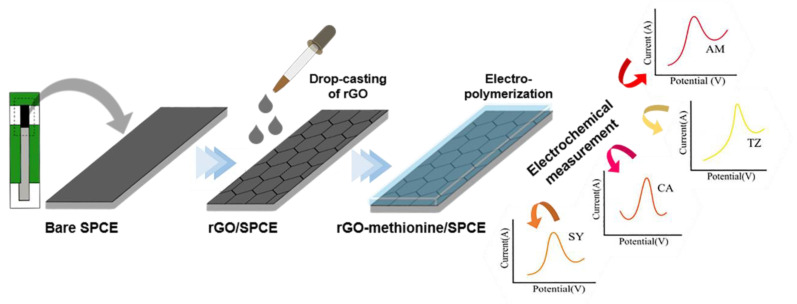
Schematic representation of modification process of rGO-methionine/SPCE.

The synthesized rGO was modified on SPCE by drop-casting. Firstly, 1 mg of pre-pared rGO was dispersed in 1 mL of 30% ethanol and sonicated for 30 min. Then, 3 µL of well-dispersed rGO was dropped directly on the surface of SPCE. The rGO modified SPCE was dried in a desiccator for 2 h. Poly(l-methionine) film was modified on rGO modified SPCE by electrochemical polymerization. CV was carried out in PBS pH 7.0 containing 2.5 mM of l-methionine with potential range of −0.6 to 2.0 V at scan rate of 100 mV/s for 3 cy-cles. After polymerization, the rGO-methionine modified SPCE was rinsed with DI water and dried overnight in the desiccator. Schematic representation of modification process is illustrated in Scheme 2.

## 4. Conclusions

In conclusion, we reported the electrochemical determination of amaranth, tartrazine, sunset yellow, and carminic acid by SPCE modified with rGO and methionine film. With the synergistic effect of rGO and methionine film, the modified sensor significantly enhanced the catalytic activity, exhibited wide linear range, and demonstrated low detection limit of these 4 dyes. Moreover, the modified sensor showed great performance in selectivity and repeatability as well as in real sample analysis. Thus, the proposed sensor can be a good alternative for food dyes determination.

## Figures and Tables

**Table 1:** Electrochemical response of amaranth, tartrazine, sunset yellow, and carminic acid on bare SPCE, rGO/SPCE, and rGO-methionine/SPCE.

Dye	Electrode	Dye Concentration (µM)	Oxidative Current (µA)
Amaranth	Bare SPCE		0.09 ± 0.01
	rGO/SPCE	10	0.94 ± 0.08
	rGO-methionine/SPCE		1.05 ± 0.12
Tartrazine	Bare SPCE		0.41 ± 0.01
	rGO/SPCE	50	1.45 ± 0.20
	rGO-methionine/SPCE		3.28 ± 0.51
Sunset yellow	Bare SPCE		0.45 ± 0.04
	rGO/SPCE	50	1.52 ± 0.22
	rGO-methionine/SPCE		1.77 ± 0.37
Carminic acid	Bare SPCE		0.35 ± 0.01
	rGO/SPCE	50	1.49 ± 0.23
	rGO-methionine/SPCE		1.76 ± 0.33

**Table 2:** Comparison of the analytical performance between the developed sensor and previous electrochemical sensors of amaranth, tartrazine, sunset yellow, and carminic acid.

Dyes	Modification	Method	Electrode	Linear Range (µM)	LOD (nM)	Ref.
AM	PSS-GR-Pd/GCE	DPV	GCE	0.1–9	7	[27]
	CPE/RuO_2_/NR/DPIBr	SWV	CPE	0.008–550	3	[38]
	PLA-ERGO/GCE	DPV	GCE	0.75–75	250	[21]
	1-M-3-BIBR/CuO/SWCNTs/CPE	SWV	CPE	0.004–750	1	[39]
	GS/GCE	DPV	GCE	2500–125,000	0.75	[40]
	Au/GTA	LSV	AuE	0.3–100	100	[1]
	rGO-methionine/SPCE (This work)	DPV	SPCE	1–10 10–100	57	-
TZ	Microspheres-laccase/AuNPs/SPE	DPV	SPCE	0.2–14	40	[41]
	PGMCPE	CV	CPE	1–27 35–87	283	[22]
	g-C_3_N_4_/Graphite electrode	DPV	Graphite	0.1–10	210	[42]
	PEDOT@TbHCF/GCE	DPV	GCE	0.1–206	32	[43]
	H-SWCNT/SPCE	SWAdV	SPCE	1–8.5	60	[31]
	rGO-methionine/SPCE (This work)	DPV	SPCE	1–10 10–85	41	-
SY	MnO_2_ NRs-ERGO/GCE	LSV	GCE	0.01–2 2–10 10–100	2	[44]
	Re@CDACs/SPCE	Amperometry	SPCE	0.05–390	16	[45]
	CS/GCE	SWAdV	GCE	0.25–3.25	98	[46]
	NC-CPE	SWV	CPE	0.001–0.1	0.2	[47]
	Poly(AAm-co-EMA)/Lac/GCE	DPV	GCE	0.08–10	20	[48]
	AuNPs/PANI-co-PoAN-co-PoT/GO/AuE	SWV	AuE	5–500	14.2	[49]
	rGO-methionine/SPCE (This work)	DPV	SPCE	1–10 10–50	48	-
CA	Unmodified	DPP	DME	1–90	160	[50]
	Pd-AuNPs/Poly(Pr)/GE	SWV	Graphite	0.01–1	5.9	[34]
	rGO-methionine/SPCE (This work)	DPV	SPCE	1–20 20–60	36	-

PSS = Poly(sodium p-styrenesulfonate; GR = Graphene; Pd = Palladium nanoparticles; RuO_2_/NR = RuO2 nano-road. DPIBr = 1,3-dipropylimidazolium bromide; PLA = Poly(L-arginine); ERGO = Electrochemical reduction of graphene oxide; 1-M-3-BIBR = 1-methyl-3-butylimidazolium bromide; SWCNTs = Single-wall carbon nanotubes; GS = Graphene nanosheets; GTA = Graphene/TiO_2_-Ag based composites; AuNPs = Gold nanoparticles; PG = Poly(glycine); g-C_3_N_4_ = Graphitic carbon nitride; PEDOT@TbHCF = Poly(3,4-ethylenedioxythiophene)@Terbium hexacyanoferrate; H-SWCNT = Double-stranded copper(I) helicate-single-walled carbon nanotube; MnO_2_ NRs = MnO_2_ nanorods; Re@CDACs = Rhenium nanoparticles decorated on activated carbon; CS = Chitosan; NC = Montmorillonite nanoclay; Poly(AAm-co-EMA) = Poly(acrylamide-co-ethyl methacrylate); PANI-co-PoAN-co-PoT = Poly (aniline-co-o-anisidine-co-o-toluidine); Pd-AuNPs = Pd–Au bimetallic nanoparticles; Poly(Pr) = Polypyroline; SWV = square wave voltammetry; LSV = linear sweep voltammetry; SWV = square wave adsorption voltammetry; DPP = differential pulse polarography.

**Table 3:** Effect of interference substances on rGO-methionine/SPCE with dye concentration at 5 µM.

Substance	Variance			
	Amaranth	Tartrazine	Sunset Yellow	Carminic Acid
Without substance	100.00	100.00	100.00	100.00
Glucose 1000x	104.73	96.97	99.73	98.30
Sucrose 1000x	102.74	95.77	98.20	99.94
NaCl 100x	97.16	97.25	100.05	96.77
KCl 100x	99.14	95.82	99.62	96.48
Glycine 10x	95.30	102.53	99.93	97.43
Ascorbic acid 10x	98.48	101.49	101.51	95.95
Citric acid 10x	95.80	102.65	98.08	104.08

**Table 4:** Effect of interference substances on rGO-methionine/SPCE with dye concentration at 50 µM.

Substance	Variance			
	Amaranth	Tartrazine	Sunset Yellow	Carminic Acid
Without substance	100.00	100.00	100.00	100.00
Glucose 1000x	104.01	97.84	103.33	97.24
Sucrose 1000x	99.38	100.95	103.37	96.61
NaCl 100x	101.41	103.76	96.94	100.61
KCl 100x	104.21	99.62	99.33	102.94
Glycine 10x	99.57	98.84	98.94	103.60
Ascorbic acid 10x	98.36	97.24	98.98	104.08
Citric acid 10x	95.36	104.11	97.42	102.54

**Table 5:** Performance of modified sensor for amaranth determination in real sample.

Sample	Added (µM)	Expected (µM)	Found (µM)	Recovery (%)
Sprite	0.00		Not found	
	1.50	1.50	1.49 (1.41)	99.45 (94.02)
	2.00	2.00	2.01 (1.90)	100.44 (95.01)
	2.50	2.50	2.51 (2.41)	100.36 (96.30)
SPY classic red	0.00		0.52	
	1.50	2.02	1.95	96.44
	2.00	2.52	2.45	97.29
	2.50	3.02	3.10	102.87

**Table 6:** Performance of modified sensor for tartrazine determination in real sample.

Sample	Added (µM)	Expected (µM)	Found (µM)	Recovery (%)
Sprite	0.00		Not found	
	1.50	1.50	1.55 (1.48)	103.43 (98.34)
	2.00	2.00	2.01 (2.05)	100.48 (102.36)
	2.50	2.50	2.49 (2.40)	99.53 (96.12)
100 Plus	0.00		0.98	
	3.00	3.98	4.15	104.21
	4.00	4.98	4.91	98.46
	5.00	5.98	6.27	104.85
Sponsor lemon lime	0.00		1.82	
	4.00	5.82	5.58	95.90
	5.00	6.82	6.42	94.14
	6.00	7.82	7.89	100.82

**Table 7:** Performance of modified sensor for sunset yellow determination in real sample.

Scheme	Added (µM)	Expected (µM)	Found (µM)	Recovery (%)
Sprite	0.00		Not found	
	1.50	1.50	1.29 (1.57)	85.98 (104.76)
	2.00	2.00	1.92 (1.99)	96.12 (99.70)
	2.50	2.50	2.40 (2.48)	95.99 (99.17)
Mirinda (orange) 4 fold dilution	0.00		1.10	
	3.00	4.10	4.24	103.38
	4.00	5.10	5.18	101.50
	5.00	6.10	6.20	101.63
Fanta (orange)	0.00		2.71	
	3.00	5.71	5.71	100.01
	5.00	7.71	7.26	94.06
	7.00	9.71	8.88	91.44

**Table 8:** Performance of modified sensor for carminic acid determination in real sample.

Sample	Added (µM)	Expected (µM)	Found (µM)	Recovery (%)
Sprite	0.00	0.00		
	5.00	5.00	5.48 (4.87)	109.63 (97.40)
	10.00	10.00	11.13 (9.72)	111.29 (97.24)
	15.00	15.00	15.58 (14.28)	103.87 (95.23)
Betagan strawberry flavour	0.00		0.46	
	3.00	3.46	3.14	90.75
	4.00	4.46	4.14	92.95
	5.00	5.46	4.85	88.93
Ice-cream Nestle Eskimo (2 fold dilution)	0.00		0.95	
	3.00	3.95	3.31	83.83
	4.00	4.95	4.36	88.12
	5.00	5.95	4.86	81.66

## Data Availability

The data presented in this sudy are available on request from the corresponding author.

## Sample Availability

Not available.
